# Higher dietary inflammatory index is associated with increased all-cause mortality in adults with chronic kidney disease

**DOI:** 10.3389/fnut.2022.883838

**Published:** 2022-07-22

**Authors:** Li-Jun Yan, Fei-Ran Zhang, Chan-Shan Ma, Yang Zheng

**Affiliations:** ^1^Department of Hemodialysis, The First Affiliated Hospital of Shantou University Medical College, Shantou, China; ^2^Department of Gastroenterology Surgery, The First Affiliated Hospital of Shantou University Medical College, Shantou, China

**Keywords:** dietary inflammatory index (DII), chronic kidney disease, mortality, nutrition, national health and nutrition examination survey (NHANES)

## Abstract

**Background:**

Diet property grounded on inflammatory potential, evaluated by the dietary inflammatory index (DII), has been proven to be connected with mortality, while studies of adults with chronic kidney disease (CKD) are scarce.

**Objective:**

The purpose of this research was to evaluate the interrelationships between DII and all-cause mortality among adults with CKD.

**Methods:**

In the National Health and Nutrition Examination Survey (NHANES) 2001–2006, we identified and evaluated data of 4,554 adults with CKD. DII scores were calculated from 24 h of dietary consumption at baseline. Vital status was followed through 31 December 2015. The association of all-cause mortality with DII score was assessed using the Kaplan–Meier curve and the Cox regression analysis.

**Results:**

After an average follow-up of 132.103 months, a total of 1,246 (27.36%) deaths were recorded. The death rates in the DII tertile categories were 24.04, 26.81, and 31.23%, respectively. The Kaplan–Meier curve showed increased death risks for the high DII tertile as compared with the low DII tertile. After we adjusted for a broad range of possible confounders, the estimation between extreme tertiles of DII scores presented a positive and significant association with all-cause mortality [hazard ratio (*HR*): 1.21, 95% *CI*: 1.05–1.39].

**Conclusion:**

Our results confirm the hypothesis that proinflammatory diets contribute to the increased all-cause mortality in adults with CKD.

## Introduction

Despite ongoing progress in the therapeutic regimen for chronic kidney disease (CKD), the mortality rate of this group of patients is still high (1). Chronic systemic inflammation often occurs in people with CKD and limits overall survival (2). Diet may have a key role in the regulation of chronic inflammation, as specific food ingredients have various anti- or proinflammatory properties that can influence immunology. On this basis, the inflammatory potential of diverse nutrients was quantified using the dietary inflammatory index (DII). The DII scoring system was independent of specific population means, which contributed to its superiority (3).

Previous studies have found a link between the DII score and mortality. The MONICA/KORA Cohort demonstrated a 41% higher risk of all-cause death comparing the highest to lowest DII category (4). Another cohort with meta-analysis reported an increased risk of all-cause, cardiovascular, and cancer mortality in a population with higher DII scores (5). As a prospective cohort reported, in survivors after coronary artery bypass grafting surgery, a pro-inflammatory diet was positively associated with mortality (6). After comparing the highest to the lowest DII category in the Japanese population, a 13% increased risk of all-cause mortality was identified (7). Therefore, the association between a pro-inflammatory diet and a higher death risk was confirmed by recent reports.

To the best of our knowledge, the relationship between DII and death risk in the CKD population has not yet been stated. Therefore, the investigation of dietary quality, especially in patients suffering from CKD who may be restricted in dietary consumption, is crucial to improve the inflammatory profile, along with its impact on the death risk. Generally, the purpose of the current study is to explore the relationship between the dietary factor and all-cause mortality among the adult CKD population, which is grounded on the large U.S. nationally representative sample with a retrospective design. It is proposed that DII may be positively associated with death risk in the CKD population.

## Materials and methods

### Data collection

#### Study sample

The study data originating from the National Health and Nutrition Examination Survey (NHANES) (RRID:SCR_013201) 2001–2006 were combined to increase the sample size. The NHANES was sponsored by the National Center for Health Statistics (NCHS), which used a stratified, multistage architecture to get a nationally representative sampling from the U.S. population.

In the current study, the urinary albumin-creatinine ratios (ACRs) and estimated glomerular filtration rate (eGFR) criteria were used to define CKD. ACRs were acquired from the urinary testing results and classified as less than 30, 30–300, or greater than 300 mg/g. The eGFR was calculated using the isotope dilution mass spectrometry 4-variable Modification of Diet in Renal Disease Study equation (MDRD)(8). CKD stages were categorized according to the following scale: stage 1, ACR ≥ 30 mg/g, along with eGFR > 90 mL/min/1.73 m^2^; stage 2, ACR ≥ 30 mg/g, along with 60 ≤ eGFR < 89 mL/min/1.73 m^2^; stage 3, 30 ≤ eGFR < 59 mL/min/1.73 m^2^; stage 4, 15 ≤ eGFR < 29 mL/min/1.73 m^2^; and stage 5, eGFR < 15 mL/min/1.73 m^2^ (9).

There were a total of 31,509 samples included from 2001 to 2006. According to the criteria, 6,330 CKD participants were selected. We identified 5,131 adults after excluding 1,199 participants less than 18 years old. After the exclusion of 570 participants with missing dietary consumption information, 5 participants with unknown death data, and 2 participants with both data missing, the final analysis enrolled 4,554 samples. The major characteristics between the final sample and participants with unavailable DII were compared (Supplementary Material 1). The NCHS’s Institutional Review Board (IRB) authorized the NHANES protocol. The ethics review from the IRB committee of our center was exempted because this study relied on publicly used, de-identified secondary data.

#### Dietary inflammatory index and mortality data

The scoring system of DII was proposed by Shivappa to assess the inflammatory gradations of 45 food parameters, which were standardized to dietary intake from representative populations around the world (3). The overall DII is calculated from the sum of individual nutrient scores from the food taken in 24 h, which includes the score coming from both the anti-inflammatory and pro-inflammatory diets. Each nutrient parameter was scored according to whether it increased (+ 1), decreased (–1), or had no effect (0) on the inflammatory biomarkers. These scores were weighted based on the study design and were called inflammatory effect scores. To avoid the arbitrariness resulting from simply using raw consumption amounts, intakes of foods and nutrition were standardized to a representative range of dietary intakes based on actual human consumption in 11 populations living in different countries across the world that provided an estimate of a mean and standard deviation (SD) for each parameter. By adding each DII score, we can achieve an individual “overall DII score.”

In this analysis, we used only observed intakes from the first 24-h dietary recall. The first 24-h dietary consultation was held in a private space in the NHANES mobile examination center. A series of measuring tools (various circles, bean bags, glasses, thickness sticks, bowls, mugs, a ruler, household spoons, measuring cups, and spoons) were accessible in the interview room for addressing the quantity of dietary intake. In total, thirty-three available kinds of food components from the NHANES dataset were used to obtain the overall DII, which includes energy, ethanol, vitamin B12/B6, fiber, magnesium, total fat, monounsaturated fatty acids, b-carotene, caffeine, niacin, docosapentaenoic (22:5), eicosatetraenoic (20:4), octadecatetraenoic (18:4), octadecatrienoic (18:3), eicosapentaenoic (20:5), docosahexaenoic (22:6), octadecadienoic (18:2), protein, polyunsaturated fatty acids, folic acid, selenium, iron, thiamin, carbohydrate, cholesterol, vitamins A/C/D/E, riboflavin, saturated fat, and zinc. The more negative DII level represents a more anti-inflammatory dietary intake, whereas the increased score reflects a more proinflammatory dietary intake.

Mortality was defined as a binary variable for alive or dead. The mortality data were obtained from the National Death Index (NDI) files, which were linked to the NHANES dataset through 31 December 2015. A total of 12 characteristics (such as date of birth, sex, and social security number) were acquired to connect the NHANES samples with the NDI to confirm survival status. The follow-up period was calculated from the interview to the happening of the death event or the end of 2015. Due to massive data missing regarding the specific cause of death, we use only all-cause mortality in our analysis.

#### Covariates

If the covariates could shift the estimates of DII on mortality exceeding 10% or had previously reported association with mortality in the CKD population, they were adopted in the fully adjusted models. A series of covariates were acquired accordingly: basic demographic information, such as age at baseline, sex, and race; personal characteristics and underlying diseases, such as waist circumference, body mass index (BMI), physical activity, congestive heart failure, coronary heart disease, stroke, chronic kidney disease, and hypertension; laboratory detection data, such as urinary albumin, urinary creatinine, albumin, phosphorus, serum glucose, serum vitamin B12, hemoglobin, uric acid, and inferred data eGFR. The race was designated as Mexican American, Non-Hispanic white, Non-Hispanic black, other Hispanic, and other races. Physical activity was categorized into four grades according to the level of activity intensity: sit during the day, stand/walk a lot, light load/climb stairs often, and heavy work/load. Respondents who answered yes to the following questions were classified as being diagnosed with the corresponding disease: “Have you ever been informed by a health professional or a doctor that you had hypertension/congestive heart failure/stroke/chronic kidney disease/coronary heart disease?” The missing values regarding waist circumference (*n* = 224), BMI (*n* = 167), urinary albumin (*n* = 85), urinary creatinine (*n* = 85), hemoglobin (*n* = 176), serum vitamin B12 (*n* = 246), albumin (*n* = 264), serum glucose (*n* = 264), phosphorus (*n* = 264), and uric acid (*n* = 264) were imputed with median.

### Statistical analysis

Continuous variates are indicated as mean ± standard deviation (SD) or median (range) according to data distribution, while categorical variates given as frequencies and percentages. The Kruskal–Wallis *H*-test (skewed distribution), one-way ANOVA test (normal distribution), and χ^2^ (categorical variables) were applied to evaluate variance among different DII (tertiles). The Kaplan–Meier method was used to estimate the survival among different DII levels, and any differences in survival were evaluated with a stratified log-rank test. The study population was censored on 31 December 2015 if they survived the thorough follow-up cycle. The univariate and multivariate Cox proportional-hazards regression models were applied to analyze the relationship between DII and all-cause mortality with three different models. Therefore, no covariates were adjusted in model 1, and only socio-demographic variables were adjusted in model 2, while the covariates presented in Table 1 were fully adjusted in model 3. To test the robustness of our results, we converted DII into a categorical variable according to tertile and calculated the *p* for trend to confirm the results of DII as the continuous variable, along with the examination of non-linear possibility.

**TABLE 1:** Baseline characteristics of the study participants with chronic kidney disease (CKD) (*n* = 4,554).

Characteristics	Dietary inflammatory index			*P*-values
	Low (–4.55 to 0.63)	Middle (0.64–2.07)	High (2.08–4.44)	
N	1,518	1,518	1,518	
Age (year)	53.93 (19.48)	54.47 (19.72)	55.62 (19.99)	0.055
BMI (kg/m^2^)	28.35 (6.49)	28.83 (7.05)	28.77 (6.76)	0.102
Waist circumference (cm)	97.52 (15.87)	97.99 (15.03)	97.60 (15.20)	0.671
eGFR (mL/min/1.73 m^2^)	57.75 (24.88)	57.76 (24.28)	55.80 (21.54)	0.031
Urinary albumin (μg/mL)	9.60 (4.30–33.38)	11.45 (5.30–44.80)	11.80 (5.50–40.88)	< 0.001
Urinary creatinine (g/L)	0.99 (0.53–1.49)	1.06 (0.63–1.56)	1.04 (0.63–1.63)	< 0.001
Hemoglobin (g/dL)	14.02 (1.51)	13.88 (1.52)	13.73 (1.47)	< 0.001
Albumin (g/dL)	4.17 (0.35)	4.12 (0.36)	4.12 (0.34)	< 0.001
Serum VitB12 (pg/mL)	487.50 (375.25–666.50)	472.00 (355.25–609.50)	472.00 (348.25–592.75)	< 0.001
Serum glucose (mg/dL)	101.34 (38.18)	103.32 (42.23)	102.23 (40.37)	0.399
Phosphorus (mg/dL)	3.80 (0.55)	3.79 (0.53)	3.80 (0.52)	0.531
Uric acid (mg/dL)	5.21 (1.46)	5.31 (1.50)	5.30 (1.48)	0.151
Time to death or censorship from interview (months)	135.97 (41.18)	132.53 (44.15)	127.82 (46.60)	< 0.001
Gender				< 0.001
Female	960 (63.24%)	1,048 (69.04%)	1,164 (76.68%)	
Male	558 (36.76%)	470 (30.96%)	354 (23.32%)	
Stroke				< 0.001
Yes	65 (4.28%)	70 (4.61%)	117 (7.71%)	
No	1,392 (91.70%)	1,377 (90.71%)	1,322 (87.09%)	
No records	61 (4.02%)	71 (4.68%)	79 (5.20%)	
Coronary heart disease				0.477
Yes	88 (5.80%)	101 (6.65%)	94 (6.19%)	
No	1,360 (89.59%)	1,330 (87.62%)	1,338 (88.14%)	
No records	70 (4.61%)	87 (5.73%)	86 (5.67%)	
Congestive heart failure				0.253
Yes	68 (4.48%)	71 (4.68%)	88 (5.80%)	
No	1,382 (91.04%)	1,367 (90.05%)	1,346 (88.67%)	
No records	68 (4.48%)	80 (5.27%)	84 (5.53%)	
Hypertension				0.017
No	920 (60.61%)	865 (56.98%)	838 (55.20%)	
Yes	593 (39.06%)	642 (42.29%)	674 (44.40%)	
No records	5 (0.33%)	11 (0.72%)	6 (0.40%)	
Physical activity				< 0.001
Sit during the day	340 (22.40%)	400 (26.35%)	523 (34.45%)	
Stand/walk a lot	383 (25.23%)	430 (28.33%)	398 (26.22%)	
Light load/climb stairs often	258 (17.00%)	265 (17.46%)	201 (13.24%)	
Heavy work/load	447 (29.45%)	337 (22.20%)	330 (21.74%)	
No records	90 (5.93%)	86 (5.67%)	66 (4.35%)	
Race				< 0.001
Black	241 (15.88%)	309 (20.36%)	369 (24.31%)	
Mexican_American	335 (22.07%)	315 (20.75%)	283 (18.64%)	
Other_Hispanic	48 (3.16%)	55 (3.62%)	64 (4.22%)	
Other_race, ethnicity	894 (58.89%)	839 (55.27%)	802 (52.83%)	
Chronic kidney disease				0.430
Stage1	137 (9.03%)	134 (8.83%)	108 (7.11%)	
Stage2	184 (12.12%)	207 (13.64%)	202 (13.31%)	
Stage3	1,144 (75.36%)	1,130 (74.44%)	1,155 (76.09%)	
Stage4	41 (2.70%)	35 (2.31%)	45 (2.96%)	
Stage5	12 (0.79%)	12 (0.79%)	8 (0.53%)	
Mortality				< 0.001
Alive	1,153 (75.96%)	1,111 (73.19%)	1,044 (68.77%)	
Death	365 (24.04%)	407 (26.81%)	474 (31.23%)	

Values are presented as mean (standard deviation, SD) or median (Q1–Q3) for continuous variables, and as number (percentage) for categorical variables. BMI, body mass index; eGFR, estimated glomerular filtration rate.

All the statistical analyses were performed with software EmpowerStats (X&Y Solutions, Inc., Boston, MA)^1^ and software R (The R Foundation, RRID:SCR_001905).^2^ The results were affirmed statistically significant if *p*-values were less than 0.05 (two-sided).

## Results

The median DII score was 1.464 (0.152–2.382). After analyzing 4,554 participants, we found a mean follow-up period of 132.103 months with a maximum of 181 months. In total, 1,246 (27.36%) death events were recorded. The baseline characteristics of the study cohort are presented in Table 1. Globally, samples with higher DII scores are more prone to be female and to have lower levels of physical activity. The prevalence of hypertension and stroke was higher in participants in the high DII tertile, as these basic diseases were correlated with the DII score. Besides, samples with higher DII levels are more prone to have higher values in urinary albumin, urinary creatinine, with lower values in eGFR, hemoglobin, albumin, and serum vitamin B12. Moreover, the composition of race is also different among the tertiles.

**FIGURE 1:**
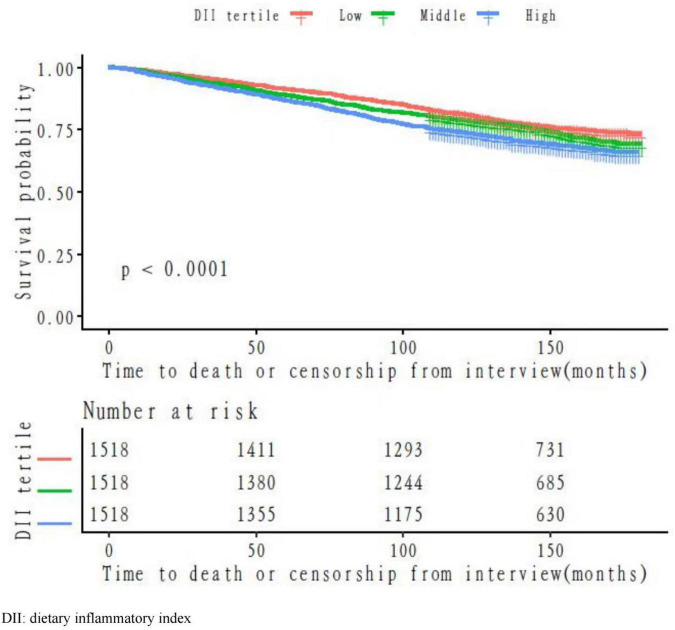
Survival probability by the level of the dietary inflammatory index (DII).

To illustrate the propensity of mortality hazards across the levels of DII per time, the Kaplan–Meier survival functions stratified by different levels of DII were presented in Figure 1. The high tertile of DII had the lowest overall survival benefit when compared with the other tertiles (*p* < 0.05 for the log-rank test).

In this retrospective cohort, we observed an association between the DII score and all-cause death risk (Table 2). The analyses on continuous DII score revealed a 9% (95% *CI*: 1.05–1.13) higher hazard of death with each 1-unit growth of the DII score, without other confounders adjusted. The death risk persisted when confounders were fully adjusted (*HR*: 1.05, 95% *CI*: 1.01–1.09). The death rates in the DII tertile categories were 24.04, 26.81, and 31.23%, respectively. Participants with the highest DII score were prone to gain greater risk of death as compared with those with the lowest tertile (*HR*: 1.38, 95% *CI*: 1.21–1.59), without other confounders adjusted. The association persisted after potential confounders were further controlled (*HR*: 1.21, 95% *CI*: 1.05–1.39). Additionally, analyses with DII tertile as a continuous variable showed a significant *p* for the trend in the adjusted model (*p*_trend_ = 0.0081).

**TABLE 2:** Multivariate analysis of the dietary inflammatory index associated with all-cause mortality, stratified by potential effect modifiers.

	Total individuals (No. of deaths)	HR (95% CI), P	
Dietary inflammatory index			
Low	1,518 (366)	1.0	
Middle	1,518 (407)	1.13 (0.98, 1.31) 0.0847	
High	1,518 (473)	1.21 (1.05, 1.39) 0.0077	
Ptrend		0.0081	
Continuous (1-unit increment)	4,554 (1,246)	1.05 (1.01, 1.09) 0.0104	
Stratification analysis			P interaction
Age (year)			0.0278
< 65	2,909 (306)	1.14 (1.05, 1.23) 0.0008	
≥65	1,645 (940)	1.03 (0.99, 1.08) 0.1267	
Gender			0.8377
Female	3,172 (701)	1.05 (1.00, 1.11) 0.0440	
Male	1,382 (545)	1.05 (0.99, 1.10) 0.0016	
Body mass index (kg/m^2^)			0.6876
< 25	1,383 (380)	1.03 (0.96, 1.10) 0.4542	
≥25, < 30	1,657 (514)	1.07 (1.01, 1 .13) 0.0316	
≥30	1,514 (352)	1.05 (0.98, 1.13) 0.1287	
Waist circumference tertile (cm)			0.5844
< 91.2	1,515 (314)	1.02 (0.95, 1.09) 0.5864	
≥ 91.2, < 102.6	1,515 (499)	1.05 (0.98, 1.11) 0.1586	
≥102.6	1,524 (433)	1.07 (1.01, 1.14) 0.0287	
Hemoglobin tertile (g/dL)			0.1251
<13.4	1,508 (435)	1.04 (0.97, 1.11) 0.2810	
=13.4, < 14.5	1,514 (404)	1.01 (0.95, 1.08) 0.6975	
=14.5	1,532 (407)	1.11 (1.04, 1.18) 0.0013	
Albumin tertile (g/dL)			0.0528
<4.0	1,145 (382)	1.00 (0.93, 1.07) 0.9262	
≥4.0, < 4.3	1,739 (484)	1.11 (1.05, 1.18) 0.0007	
≥4.3	1,670 (380)	1.03 (0.96, 1.10) 0.3970	
Serum glucose (mg/dL)			0.5434
≥108	3,699 (872)	1.05 (1.01, 1.10) 0.0198	
>108	855 (374)	1.03 (0.96, 1.10) 0.4408	
Urinary albumin tertile (μg/mL)			0.9455
<6.8	1,512 (249)	1.04 (0.97, 1.13) 0.2560	
≥6.8, < 23.2	1,524 (401)	1.06 (0.99, 1.13) 0.0990	
≥23.2	1,518 (596)	1.04 (0.99, 1.10) 0.1210	
Urinary creatinine tertile (g/L)			0.7725
<0.74	1,495 (442)	1.07 (1.01, 1.14) 0.0241	
≥0.74, < 1.35	1,530 (497)	1.04 (0.98, 1.10) 0.1954	
≥1.35	1,529 (307)	1.06 (0.98, 1.14) 0.1587	
Serum VitB12 tertile (pg/mL)			0.9092
<399	1,514 (418)	1.06 (0.99, 1.13) 0.0968	
≥399, < 560	1,522 (408)	1.06 (0.99, 1.13) 0.0826	
≥560	1,518 (419)	1.04 (0.98, 1.11) 0.1747	
Phosphorus tertile (mg/dL)			0.4681
<3.6	1,380 (401)	1.04 (0.98, 1.12) 0.1865	
≥3.6, < 4.0	1,561 (434)	1.09 (1.02, 1.16) 0.0113	
≥4.0	1,613 (411)	1.03 (0.96, 1.09) 0.3981	
Uric acid tertile (mg/dL)			1.0000
<4.6	1,469 (250)	1.03 (0.97, 1.08) 0.3170	
≥4.6, <5.7	1,502 (388)	1.06 (1.00, 1.12) 0.0507	
≥5.7	1,583 (608)	1.03 (0.98, 1.08) 0.1936	
Race			0.3041
Black	919 (225)	1.09 (0.99, 1.20) 0.0658	
Mexican_American	933 (176)	0.99 (0.90, 1.10) 0.9175	
Other Hispanic	167 (24)	0.87 (0.63, 1.21) 0.4066	
Other race, ethnicity	2,535 (821)	1.08 (1.03, 1.13) 0.0019	

Models were adjusted for race, waist circumference, stroke, coronary heart disease, congestive heart failure, eGFR, urinary albumin, urinary creatinine, hemoglobin, serum vitamin B12, albumin, serum glucose, phosphorus, uric acid, hypertension, physical activity, chronic kidney disease, except for the stratification factor itself.

The subgroup analyses, stratified by potential effect modifiers, did not reveal prominent differences regarding the impact of DII on all-cause death, except for in the categories of age (< 65 vs. ≥ 65 years) (Table 2). DII contributed more to the death risk in adults less than 65 years of age than in the elderly population (*HR*: 1.14, 95% *CI*: 1.05–1.23 vs. 1.03, 95% *CI*: 0.99–1.08), with *p* interaction < 0.05.

## Discussion

In this large retrospective cohort with an average follow-up of 132.03 months, we observed a link between DII and all-cause death risk in a large CKD population of U.S. adult. As the result presented, persons with higher DII scores are at a more obvious risk of death. These associations are still obtained after adjusting for potential confounders, especially in populations less than 65 years. The difference between age stratifications can be partially attributable to the higher incidence of various comorbidities and shorter follow-up duration (146.89 ± 31.55 vs. 105.96 ± 50.66 m) in the elder subgroup.

Previous reports had illustrated the link between dietary-induced inflammation and the growing risk of various physical disorders, such as cardiovascular disease (10, 11), diabetes (12), cancer (13), and CKD (14). There was evidence that pro-inflammatory components in dietary intake might have an impact on CKD development, by promoting tissue-specific and systemic metabolic dysfunction (15).

Although the relation between dietary inflammatory potential and the risk of new-onset renal dysfunction had been reported in a series of studies (16, 17), the link between DII and mortality in the CKD population is a relatively novel area of research. The results obtained in this study provide support to the hypothesis that inflammatory injury may address the association between poor dietary consumption and increased death risk in the CKD population.

Studies with respect to DII, which had been carried out in other populations, address similar conclusions to our results. Supporting our findings, a prospective cohort study with the U.S. population demonstrated that individuals who consumed a more pro-inflammatory diet were at an increased risk of dying from all-cause (34%), cardiovascular disease (46%), and cancer (46%) compared with individuals who consumed a more anti-inflammatory diet (18). Similar to our cohort, the study was based on a single self-reported 24 h recall, which may not be an adequate reflection of the usual diet. In a meta-analysis including 38 studies, a higher level of DII was associated with a higher risk for mortality caused by all types of cancer by 16% [odds ratio (OR): 1.16; 95% *CI*: 1.01–1.32] (19). Although the reviewed studies were heterogeneous in terms of population characteristics, design, and duration of follow-up periods.

To date, the potential mechanisms remain uncertain about the issue that higher DII contributes to increased mortality. However, some feasible considerations with biological plausibility have been proposed. The underlying mechanisms include increased incidence of obesity and metabolic syndrome (20), the turbulence of glucose and insulin metabolism (21), and shortening of telomere length (22) accompanied by higher DII levels.

Recently, the pieces of evidence have proposed that intestinal microbiota and their metabolites may influence the progression and prognosis of CKD (23, 24). For example, as the degradative product of choline and L-carnitine by intestinal microbiota, trimethylamine oxide is proven to be highly associated with the death risk in CKD (25). In addition, the composition of intestinal microbiota may be noticeably impacted by the dietary intake (26). The DII derived from dietary intake may influence the mortality in CKD by modulating the composition of intestinal microbiota. We may achieve some valuable information if we focus on the dietary inflammatory potential of patients with CKD and its relation to microbiota.

This cohort has the largest sample focusing on the association of DII with mortality in the adult CKD population. The results can be extended to the general U.S. population due to the randomly sampling design. Since data acquisition was carried out on all days of the week from the NHANES dataset, the possibility of day-specific information bias is fairly low. There are some limitations to our study as well. First, the major limitation is the lack of a specific cause of death, which does not allow to come to accurate inferences on the issue. Second, merely a single 24-h dietary recall is not sufficient in reflecting a person’s long-term conventional eating habits (27). Within-individual random measurement errors cannot be excluded when using only observed intakes from a 24-h dietary recall. Third, the calculation of DII scores was based on the accessible 33 out of 45 dietary components, which brings potential interference with our findings. Fourth, in the setting of an observational study, we cannot exclude the possibility that some of our results were affected by residual confounding, even with various covariates adjusted. Finally, according to the data presented in Supplementary Material 1, populations with missing DII were more prone to lose information on waist circumference and BMI, with a large portion of laboratory tests undetected, which have a higher risk of death (46.84 vs. 27.36%) in shorter follow-up duration (136 vs. 145 m). Given the consideration of various different characteristics between the final and the withdrawn samples, the exclusion of the withdrawn samples may lead to potential bias in the results.

## Conclusion

In summary, a healthy and appropriate dietary consumption with a lower DII score was inversely associated with all-cause mortality among the adult CKD population. These findings support recommendations for the adult CKD population to follow a balanced diet with lower inflammatory potential. Dietary instruction might provide a modifiable measure for CKD management, while more investigations are necessary to figure out the potential mechanism.

## Data availability statement

Publicly available datasets were analyzed in this study. This data can be found here: https://www.cdc.gov/nchs/nhanes/index.htm.

## Author contributions

L-JY, F-RZ, and YZ designed the research and wrote the article. L-JY and YZ conducted the research. F-RZ and C-SM analyzed the data. F-RZ and YZ had primary responsibility for the final content. All authors have read and approved the final version of the manuscript for publication.

## Conflict of interest

The authors declare that the research was conducted in the absence of any commercial or financial relationships that could be construed as a potential conflict of interest.

## Publisher’s note

All claims expressed in this article are solely those of the authors and do not necessarily represent those of their affiliated organizations, or those of the publisher, the editors and the reviewers. Any product that may be evaluated in this article, or claim that may be made by its manufacturer, is not guaranteed or endorsed by the publisher.

## Footnotes

1
http://www.empowerstats.com

2
http://www.R-project.org
