# Supplementary material for: Higher dietary inflammatory index is associated with increased all-cause mortality in adults with chronic kidney disease
Source: Front Nutr. 2022 Jul 22;9:883838. doi: 10.3389/fnut.2022.883838 (PMC9355393; doi:10.3389/fnut.2022.883838)
Supplement: Supplementary file 1 [file Table_1.DOCX]

Supplement material 1

Comparison of characteristics between final samples and participants with missing dietary information

|  | Final samples | Missing dietary data group | P-value |
| --- | --- | --- | --- |
| N | 4554 | 570 |  |
| Age(yr) | 54.67 (19.74) 56.00 (39.00-71.00) | 62.03 (21.02) 67.00 (45.00-82.00) | <0.001 |
| eGFR(ml/min/1.73m2) | 57.10 (23.62) 52.41 (45.14-59.04) | 50.49 (16.73) 50.30 (41.43-55.95) | <0.001 |
| Time to death or censorship from interview(months) | 132.10 (44.15) 145.00 (117.00-164.00) | 111.77 (57.54) 136.00 (62.00-160.00) | <0.001 |
| Coronary heart disease |  |  | 0.050 |
| Yes | 283 (6.51%) | 44 (7.97%) |  |
| No | 4028 (92.60%) | 498 (90.22%) |  |
| No records | 39 (0.90%) | 10 (1.81%) |  |
| Stroke |  |  | <0.001 |
| Yes | 252 (5.79%) | 55 (9.96%) |  |
| No | 4091 (94.05%) | 496 (89.86%) |  |
| No records | 7 (0.16%) | 1 (0.18%) |  |
| Congestive heart failure |  |  | 0.006 |
| Yes | 227 (5.22%) | 47 (8.51%) |  |
| No | 4095 (94.14%) | 502 (90.94%) |  |
| No records | 28 (0.64%) | 3 (0.54%) |  |
| Gender |  |  | 0.047 |
| Female | 3172 (69.65%) | 420 (73.68%) |  |
| Male | 1382 (30.35%) | 150 (26.32%) |  |
| Mortality |  |  | <0.001 |
| Alive | 3308 (72.64%) | 303 (53.16%) |  |
| Death | 1246 (27.36%) | 267 (46.84%) |  |
| Hypertension |  |  | 0.663 |
| No | 2623 (57.88%) | 321 (56.91%) |  |
| Yes | 1909 (42.12%) | 243 (43.09%) |  |
| Physical activity |  |  | <0.001 |
| Sit during the day | 1263 (29.29%) | 273 (50.28%) |  |
| Stand/Walk a lot | 1211 (28.08%) | 110 (20.26%) |  |
| Light load/Climb stairs often | 724 (16.79%) | 59 (10.87%) |  |
| Heavy work/load | 1114 (25.83%) | 101 (18.60%) |  |
| Race |  |  | 0.027 |
| Black | 919 (20.18%) | 101 (17.72%) |  |
| Mexican_American | 933 (20.49%) | 97 (17.02%) |  |
| Other_Hispanic | 167 (3.67%) | 17 (2.98%) |  |
| Other_Race,ethnicity | 2535 (55.67%) | 355 (62.28%) |  |
| Chronic kidney disease |  |  | <0.001 |
| Stage1 | 379 (8.32%) | 20 (3.51%) |  |
| Stage2 | 593 (13.02%) | 32 (5.61%) |  |
| Stage3 | 3429 (75.30%) | 494 (86.67%) |  |
| Stage4 | 121 (2.66%) | 15 (2.63%) |  |
| Stage5 | 32 (0.70%) | 9 (1.58%) |  |

Mean(SD) Median (Q1-Q3) / N(%)

Populations with missing dietary data were more prone to conceal information on waist circumference and BMI, with large portion of laboratory tests undetected. In the missing dietary data group, there were lots of data non-acquirable including waist circumference(n=404), BMI(n=365), urinary albumin(n=358), urinary creatinine(n=358), hemoglobin(n=368), serum VitB12(n=373), albumin(n=376), serum glucose(n=376), phosphorus(n=376) and uric acid(n=376).
